# Artificial neural network prediction of performance and emissions of a diesel engine fueled with palm biodiesel

**DOI:** 10.1038/s41598-022-13413-9

**Published:** 2022-06-03

**Authors:** A. S. El-Shafay, Umar F. Alqsair, S. M. Abdel Razek, M. S. Gad

**Affiliations:** 1 Department of Mechanical Engineering, College of Engineering, Prince Sattam Bin Abdulaziz University, Alkharj, 16273 Saudi Arabia; 2 Mechanical Power Engineering Department, Faculty of Engineering, Mansoura University, Mansoura, 35516 Egypt; 3 Mechanical Engineering Department, Faculty of Engineering, Misr University for Science and Technology, 6th October City, Egypt; 4 Mechanical Engineering Department, Faculty of Engineering, Fayoum University, Fayoum, Egypt

**Keywords:** Biotechnology, Hydrology

## Abstract

Increasing of energy consumption, depletion of petroleum fuels and harmful emissions have triggered the interest to find substitute fuels for diesel engines. Palm ethyl ester was synthesized from palm oil through transesterification process. The physicochemical properties of palm biodiesel have been measured and confirmed in accordance with ASTM standards. The aim of the paper is to show the effect of different diesel-palm biodiesel blends on performance, combustion and emissions in diesel engine at engine load variation. Artificial Neural Network was used for the prediction of engine performance, exhaust emission and combustion characteristics parameters. Palm ethyl ester and diesel oil were blended in 5, 10, 15 and 20 by volume percentage. The maximum decreases in thermal efficiency, fuel–air equivalence ratio for B20 were 1.5, 3.5, 6 and 8% but the maximum increases in BSFC, exhaust gas temperature and NO_x_ emission for B20 at full load about diesel fuel were 9, 8 and 10%, respectively. The highest decreases in CO, HC and smoke emissions of B20 about diesel oil at full load were 2, 35 and 18.5% at full load, respectively. Biodiesel blend B20 achieved the maximum declines in peak HRR, cylinder temperature and combustion duration about diesel fuel. The results of ANN were compared with experimental results and showed that ANN is effective modeling method with high accuracy. Palm biodiesel blends up to 20% showed the highest enhancements in engine performance, combustion and emission reductions compared to diesel fuel.

## Introduction

Environmental concern and fossil oil depletion have caused the interest search about substitute fuels in diesel engine^1–4^. The growing interest in converting agricultural and industrial residues into renewable energy sources as well as environmental impact is established nowadays^5^. Biodiesel is one solution for these problems^6,7^. Biodiesel is biodegradable, non-toxic, renewable and free of sulfur and aromatics^8–11^. Due to the lower volatility, higher density and higher viscosity of vegetable oils, so, compression ignition engines cannot be run with these oils. Poor atomization and spray penetration of fuel were happened due to its properties. Spray penetration issues were caused by the lubrication oil thickening, engine deposits and piston ring sticking. Oil properties can be enhanced through a transesterification process. Biodiesel produces fewer emissions compared to diesel fuel^12–15^. The temperature was dropped by the increase of additive volume fraction and this leads the effect on thermal conductivity and heat distribution in the fuel^16^.

Several researchers examined biodiesel blends and found that as the biodiesel content increase, the peak cylinder pressure inside the combustion chamber was decreased. Lower burned fuel in the premixed combustion stage, higher biodiesel percentages resulted in lower heat release rate^17–19^. Vegetable oils were used in its neat form from Palm, Pongamia, Jatropha, Neemseed oil and Fish oil^20^. When compared to other oils, the palm tropical plant has the highest oil yield of 5000 kg/hectare. Palm oil demonstrates good competitiveness among other crops for biodiesel production. Palm oil mixed with diesel oil has emerged as substitute fuel^21^. When compared to diesel oil, palm biodiesel produced less engine power while consuming more fuel^22,24^. According to Bari et al., utilization of crude palm oil in diesel engine increased the specific fuel consumption by 26%^25^. In comparison to diesel fuel, palm biodiesel emits less HC and CO emissions^26,27^. Deepanraj et al.^28^ stated that brake thermal efficiencies of palm biodiesel mixtures as B10, B20, B30, B40 and B50 were decreased about diesel fuel and associated with the output increase. CO and HC concentrations were declined than diesel fuel. Furthermore, NOx production was higher than diesel fuel and increased with the biodiesel percentage increase.

Kinoshita et al.^29^ recorded that palm biodiesel blended fuels produced less BTE while BSFC was higher in comparison to pure diesel. Reductions in HC and smoke emissions were shown about diesel fuel. Abdul et al.^30^ investigated that diesel and palm oil blends of 2, 5 and 10% produced the deviations in cylinder pressure and reductions in heat release rate and ignition delay compared to diesel oil. Kalam et al.^31^ stated that brake power produced from crude palm methyl ester B100 was lower about diesel fuel. When compared to diesel fuel, BSFC was highest at B100, followed by B35 and B20. According to Sharon et al.^32^, palm ethyl ester mixtures (B25, B50, B75 and B100) showed declined BTE while BSFC were higher than diesel fuel. CO emission was lower than diesel oil. B25 produced more HC and smoke emissions than diesel fuel, whereas B50, B75, and B100 produced less.

According to Khalid et al.^33^, the output power and fuel consumption of palm ethyl ester mixtures (B5, B10, and B15) were lower about diesel fuel. In comparison to diesel oil, CO, smoke, and HC emissions were decreased. Masjuki and Prasad et al.^34^ conducted diesel engine tests with esterified palm oil. Brake power, BSFC and BTE were near to diesel oil. Vedaraman et al.^35^ reported that palm biodiesel blended fuels (B20, B30, B40 and B100) produced lower BTE than diesel fuel and it was decreased with of biodiesel percentage increase meanwhile BSFC was higher. The emissions of HC and CO were decreased about diesel oil. NO_x_ emissions were higher about crude diesel. Almeida et al.^36^ described the impact of ethyl ester on C.I.E. engine fueled with palm methyl ester B100. Pure biodiesel resulted in slightly higher BSFC about diesel oil. Moreover, CO emission was higher than diesel fuel. The diesel engine produced lower HC and greater NOx emissions than for B100 about diesel oil.

Robustness and adaptability were studied by comparing the predicted to experimental results using two training algorithms of ANN^37,38^. Non-linear mapping was accomplished by the use of multi-layer perception. Correlation coefficients of 0.995, 0.980, 0.999, 0.985, 0.980 and 0.999 were shown for exhaust gas temperature, specific fuel consumption, thermal efficiency, HC, NO_X_, smoke emissions, respectively^39^. Engine performance and emissions using ANN model are close to experimental estimates with correlation coefficient from 0.97 to 0.99^40^. Feed-forward back propagation ANN model is used to show the engine emissions and performance using palm biodiesel. ANN model was used with R > 0.99 and mean absolute error of 1.879%^41,42^. R values of validation, training and testing were 0.9994, 0.9999 and 0.9995, respectively^43^. Used ANN model algorithm is Feed-Forward Back Propagation Levenberg–Marquardt with MSE of ten neurons and three layers^44^. ANN modeling showed higher correlation coefficient values R^2^ between 0.88 and 0.95. MRE and RMSE (mean relative error and root mean square error) were both low. ANN model gave the best results of the optimization and predication of the engine emissions and performance^45^.

The above literature stated the impact of biodiesel blends on performance, combustion characteristics and emissions. Few research papers have been conducted to show the impact of biodiesel from palm oil on ignition delay, combustion duration, and cylinder temperature. These parameters were affected by the biodiesel feedstock type because of its properties. The goal of this research is to evaluate the performance, emissions, and combustion characteristics of palm biodiesel mixtures with diesel oil. Biodiesel was obtained from crude palm oil using transesterification process. Palm biodiesel blends of B5, B10, B15 and B20 were prepared. ASTM standards were met for the chemical and physical characteristics of biodiesel blends. At engine speed of 1500 rpm, the engine loading range is from zero to full load. Artificial neural network is used to predict the engine performance, emissions and combustion characteristics. The results of ANN were compared with experimental results and showed that ANN is effective modeling method with high accuracy. The prediction model is reliable with measured data. Palm biodiesel up to 20% is recommended because it shows the highest improvements in engine performance, combustion and emission reduction compared to diesel fuel.

## Materials and methods

### Biodiesel production process

**Figure 1:**
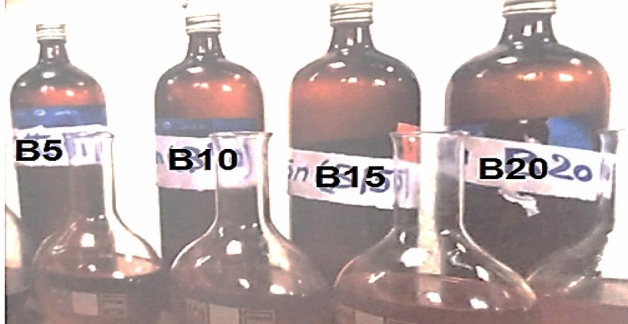
Photos of biodiesel blends samples.

A pretreatment of palm oil for biodiesel production to remove the impurities and gums by filtration and centrifugation was done. The oil moisture was eliminated by the heating of palm fatty acid distillate at 90 °C for 15 min. After the pretreatment, esterification process at which palm oil was mixed with a mixture of H_2_SO_4_ and ethanol was done. Palm oil was heated up to 80 °C for the moisture removal. The mixture of reactants is 1 L palm oil, 5 ml of sulfuric acid (H_2_SO_4_) and 250 ml of ethanol. After that, Free Fatty Acid (FFA) content was checked. If free fatty acid content becomes less than 2%, transesterification process will be used as a base catalyzed reaction produces mono alkyl esters and glycerin^46^. Blend of 250 ml ethanol and 5 gm of sodium hydroxide (NaOH) were added to the esterified oil. The oil was preheated to 65–70 °C ± 5 °C at the reaction time of 60 min. The mixture of catalyst and ethanol was stirred for 30 min and poured in oil. FFA content of mixture should be less than 0.5%. After 3 h of stirring at 700 rpm, the mixture was transferred into a separating funnel and remained to settle for 12 h. The bottom layer containing glycerin and contaminants which were eliminated and the upper layer yielded a palm ethyl ester that contained alcohol and water. Biodiesel was purified by washing it from three to four times to be purified^47,48^. A rotary evaporator was used to decrease the moisture content and obtain a pure palm ethyl ester. A diesel No. 2 was used as the reference fuel. Biodiesel blends of palm biodiesel and diesel in various volumetric proportions of 5, 10, 15, and 20% such as B5, B10, B15, and B20. Photos of biodiesel blends samples were shown in Fig. 1.

### Characterization of biodiesel from palm oil

Properties of crude diesel, palm ethyl ester and palm oil are shown in Table 1. Density of palm oil and biodiesel were greater than diesel oil. Palm biodiesel flash point was higher in comparison to diesel fuel and is safe to handle. Palm oil has higher kinematic viscosity and density than diesel fuel. Palm oil viscosity was reduced by biodiesel production process. Palm biodiesel viscosity is 4.5 cst at 40 °C. Viscosity values for diesel oil, B100 and biodiesel blends were within American Society of Testing and Materials (ASTM) standards. Palm oil has lower calorific value about diesel fuel but biodiesel value was near to crude diesel. Diesel fuel has lower cetane number than biodiesel, so, the ignition delay is lower for ethyl ester. There is no phase separation at low temperature operation because of the lower differences between the biodiesel blends in densities and viscosities values. The densities of B5, B10, B15 and B20 are 845.5, 846.5, 847 and 848 kg/m^3^. The viscosities values were 31, 27, 20 and 13.8 cSt^49^.

**Table 1:** Properties of diesel fuel, palm oil methyl ester and palm biodiesel blends.

Properties	Method	Diesel oil	Palm oil	B5	B10	B15	B20	B100
Kinematic viscosity, at 40 °C cSt	ASTMD445	3	33	31	27	20	13.8	4.5
Heating value MJ/kg	ASTMD270	42	39.5	41.85	41.8	41.75	41.7	40.5
Density, at 15 °C kg/m^3^	ASTMD1298	845	922	845.5	846.5	847	848	885
Cetane number	ASTMD613	45	39	45	45	46	46	52
Flash point °C	ASTMD92	67	230	68	70	71	73	189

The retention indices of the separated fatty acids methyl esters components were calculated using fatty acids methyl esters standards (C4–C22), (Sigma Aldrich Co.) as references. There are three main types of fatty acids that can be presented in a triglyceride which is saturated (Cn: 0), monounsaturated (Cn: 1) and polyunsaturated with two or three double bonds (Cn: 2, 3). The major fatty acids in palm biodiesel were oleic, linoleic, palmitic and the stearic fatty acid. GC–MS analysis shows constituents as Myristic (1.06%), Palmitic (39.2%), Oleic (43.4%), Linoleic (10.1%) and Stearic (4.8%) (see Table 2).

**Table 2:** Chemical composition of palm biodiesel by GC–MS analysis.

Fatty acid composition	Percentage
Lauric (12:0)	0.6
Myristic (14:0)	1.06
Palmitic (16:0)	39.2
Oleic (18:1)	43.4
Arachidic (20:0)	0.04
Palmitoleic (16:1)	0.4
Linoleic (18:2)	10.1
Linolenic (18:3)	0.45
Stearic (18:0)	4.8
Gadolic (20:1)	0.13

## Experimental setup

**Figure 2:**
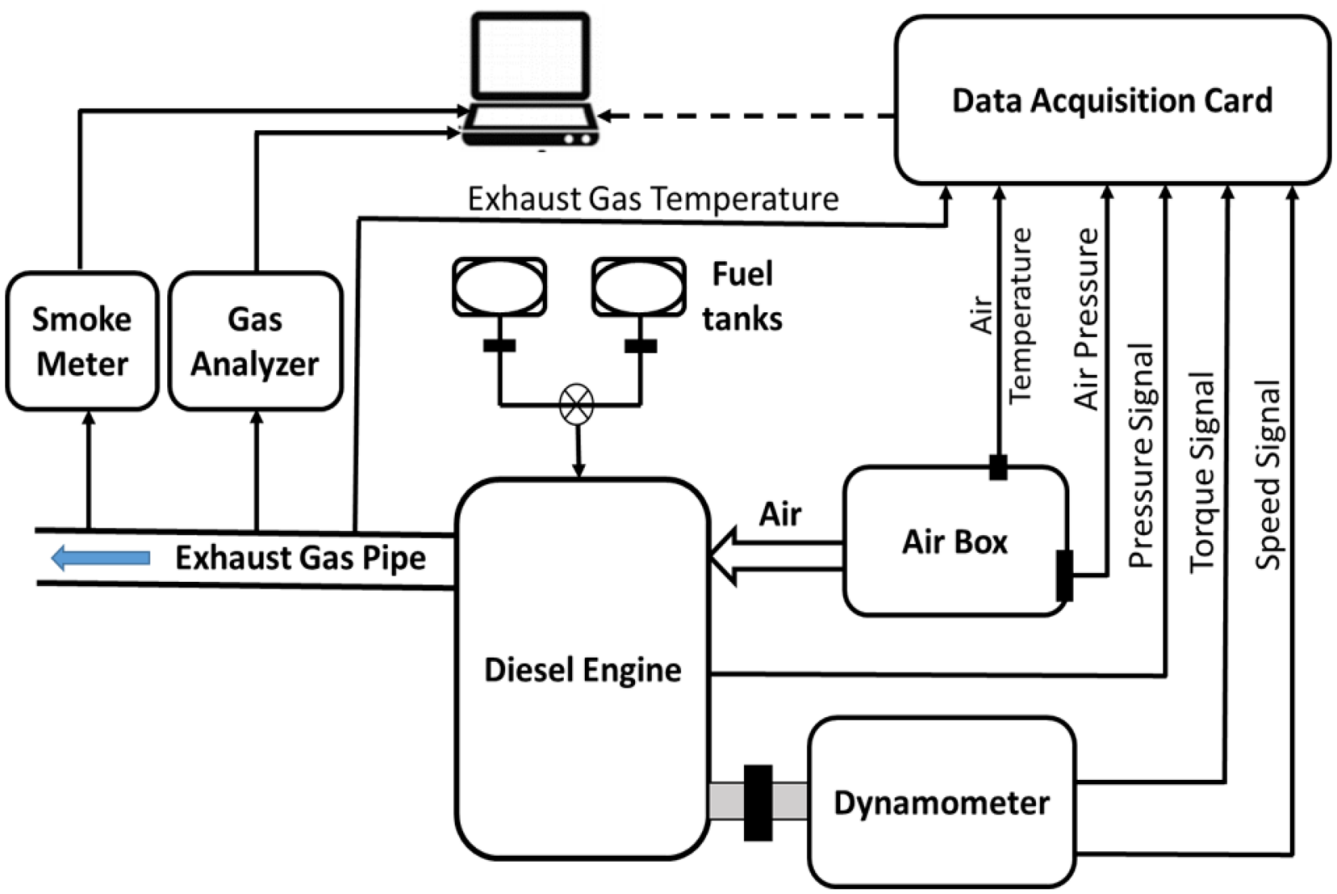
Schematic of the experimental setup.

Experiments were carried out with a diesel engine producing 5.775 kW at 1500 rpm. Used engine is single cylinder, four stroke of DEUTZ F1L511 type (Bore = 100 mm and Stroke = 105 mm and compression ratio = 17.5). Schematic diagram of test rig is shown in Fig. 2. AC generator of maximum output power of 10.5 kW was connected to the test engine to determine the engine output power. A sharp edged orifice mounted was used to evaluate the intake airflow rate. The pressure drop across the orifice was evaluated using U tube manometer. Thermocouples of type K measured the intake air and exhaust gas temperatures. A speed tachometer recorded the engine speed. Fuel consumption was measured using the consumption time of a given fuel volume of 20 ml. To estimate the cylinder pressure, a water-cooled Kistler piezoelectric pressure transducer type 601A with sensitivity of 16.5 pc/bar was employed. Cylinder pressure transducer was supported with Nexus charge amplifier of type 2692-A-0S4. To reduce the pressure signal lag and resonance, the pressure transducer was flush installed. A proximity switch identified the piston's top dead centre (TDC) (Type LM12-3004PA). The cylinder pressure data was averaged over 120 engine cycles. The data was collected using the LABVIEW program and data acquisition card (NI-USB-6210). For exhaust and smoke emissions, the MRU DELTA 1600-V gas analyzer and the OPA 100 smoke meter were utilized, respectively. The engine output power was varied from zero to 100% at a rated speed of 1500 rpm during the tests. Figure 1 depicts the experimental setup schematic diagram.

### Uncertainty analysis

All devices and sensors were calibrated. The uncertainties in (HC, NO_x_, smoke and CO emissions) and thermal efficiency were ± 1 ppm, ± 1 ppm, ± 1.5%, ± 0.01% and ± 1.5%, respectively. Output power, T_exh_, BSFC and engine speed showed the maximum uncertainties as 0.85%, 0.2%, 0.15% and 2.2%, respectively. The maximum uncertainties in cylinder pressure and TDC marking are 0.2 and 1%, respectively.

$\mathrm{Total uncertainty}=\sqrt{{(uTexh)}^{2}+{(ubp)}^{2}+{(usfc)}^{2}+{(uN)}^{2}+{(uther)}^{2}+{(uCO)}^{2}+{(uHC)}^{2}+{(uNOx)}^{2}+ {(uPcy)}^{2}+{(uTDC)}^{2}}=\sqrt{{(0.2)}^{2}+{(0.85)}^{2}+{(2.2)}^{2}+{(0.15)}^{2}+{(1.5)}^{2}+{(0.01)}^{2}+{(1)}^{2}+{(1)}^{2}+ {(0.2)}^{2}+{(1)}^{2}}$where: uT_exh_, u_bp_, u_sfc_, u_N,_ u_ther_, u_CO_, u_HC_, u_NOx_, u_pcy_, u_TDC_ are the exhaust gas temperature, Specific fuel consumption, engine speed, thermal efficiency, CO, HC, NO_x_ emissions, cylinder pressure and Top Dead Centre (TDC) proximity switch uncertainties, respectively. The total uncertainty for the whole experiment is obtained to be ± 3.3%.

## Results and discussion

### Brake thermal efficiency (BTE)

**Figure 3:**
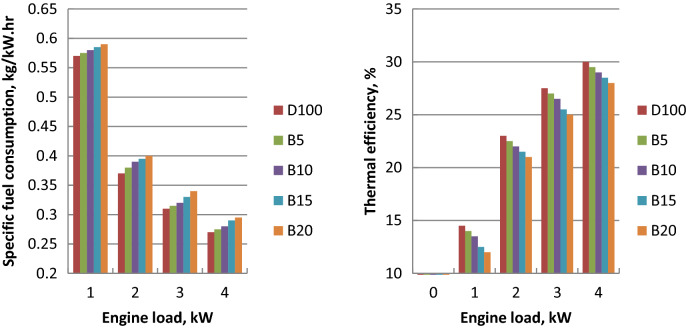
Values of BTE and BSFC against engine load for tested fuels.

Figure 3 indicates the effect of different fuels on BTE at different engine loads. Engine load increase produced the increases in thermal efficiencies. As the engine load increased, developed pumping work would be higher and result in efficient combustion. Brake thermal efficiencies for ethyl ester mixtures were lower than diesel oil at the load range. The decrease of biodiesel calorific value leads to the decrease of thermal efficiency about crude diesel. Poor volatility, improper air–fuel mixing and high viscosity led to the poor combustion characteristics of ethyl ester. The increased consumed fuel is required to compensate for the decreased calorific value in comparison to diesel oil. The maximum decreases of thermal efficiency for B5, B10, B15 and B20 were 1.5, 3.5, 6 and 8% related to diesel oil at engine load 100%. These findings are confirmed with literature^46,47^.

### Brake specific fuel consumption (BSFC)

BSFC plots of tested fuels at different engine loads were indicated in Fig. 3. Variations in brake specific fuel consumption with the brake power for all fuels revealed that as engine load increased with the fuel consumption increase. Due to its higher density and lower calorific value, BSFC of ethyl ester blends was higher than diesel fuel. Because biodiesel has a larger bulk modulus, it can discharge more fuel for the same displacement of the injection pump plunger. BSFC was increased drastically with B20 biodiesel blend. Biodiesel was associated with the problems of fuel vaporization and atomization. The higher fuel consumption is caused by the biodiesel blends low volatility, inappropriate air–fuel mixing, and higher viscosity. The highest burned fuel to produce the same brake power of biodiesel fuel is shown about diesel oil. The maximum increases in BSFC for B5, B10, B15 and B20 at full load about diesel fuel were 2, 4, 7.5 and 9%, respectively. These results are agreed with the references^46,48^.

### Exhaust gas temperature (EGT_._)

**Figure 4:**
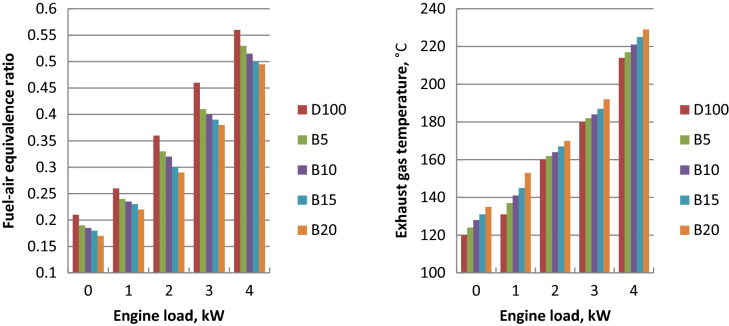
Fuel- air equivalence ratio and T_exh_ variation against engine load.

The amount of waste heat going with exhaust gases is an indication of T_exh_. Figure 4 shows the effect of tested fuels on T_exh._ at the load variation. Its increase is associated with the load increase because of the more burned fuel required to take the additional load. At all the load range, diesel fuel has the lowest exhaust gas temperature but EGT of biodiesel mixtures showed an upward trend with palm biodiesel percentage. This is owing to biodiesel blends lower calorific value, lower heat transfer rate and higher viscosity compared to diesel oil. Biodiesel was related to the increase in the exhaust heat loss. The maximum increases in exhaust gas temperature of B5, B10, B15 and B20 about diesel fuel were 2, 3, 5 and 8%, respectively. These values are confirmed with literature^47,49^.

### Air- fuel equivalence ratio (AFR)

Increased engine load resulted in the drop in air–fuel ratio due to the increase in injected fuel mass. Figure 4 depicts the fuel–air equivalence ratios of ethyl ester mixtures. Due to the increased fuel consumption and lower calorific value of biodiesel, the air–fuel ratio was reduced as the ethyl ester ratio increased in comparison to diesel fuel. Improper mixing of air and fuel, lower volatility and higher viscosity of biodiesel led to the fuel consumption increase. Biodiesel blends B5, B10, B15 and B20 showed the maximum decreases in fuel–air equivalence ratio of 5, 8, 10 and 12% about diesel fuel at full load, respectively. These findings are confirmed with the references^46,47^.

### Carbon monoxide emission (CO)

**Figure 5:**
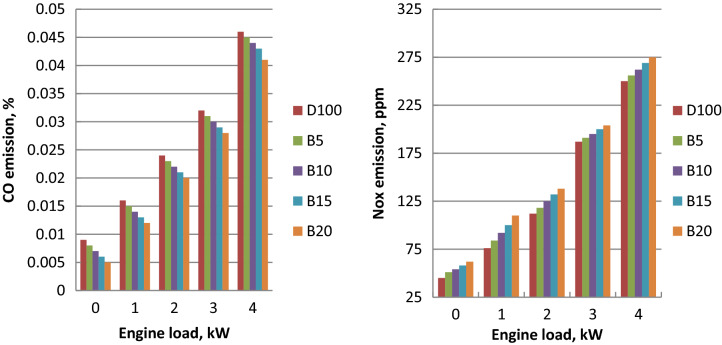
CO and NO_x_ emissions with engine load for biodiesel blends.

At the engine load variation, CO emissions of all fuels are indicated in Fig. 5. The increasing trend of CO emissions was observed with the engine output power output due to the fuel consumption increase. Carbon monoxide emission of palm ethyl ester is less than diesel fuel due to the oxygen presence and more complete oxidation. Improved combustion and air- fuel air mixing produces the decline of CO emission about diesel oil. The highest decreases in carbon monoxide emission of B5, B10 and B20 about diesel oil at full load were 2, 4, 6.5 and 11% at full load, respectively. These results are confirmed with literature^46,51^.

### Nitrogen oxides emissions (NO_x_)

NO_x_ emissions of biodiesel blends are shown as function of engine load as shown in Fig. 5. When compared to diesel fuel, fueling with biodiesel or its blends increased NOx emissions. NOx formation is produced by the higher cylinder gas temperature, lower heat transfer, and nitrogen content. Thermal NOx generation is affected by the residence time, cylinder temperature and oxygen concentration. The increased nitrogen oxide emissions were caused by the higher cetane number, shorter ignition delay, and accelerated combustion. Rise in NOx emissions is caused by the increase of cylinder temperature. At higher cylinder combustion temperatures, the dissociated nitrogen combines with the oxygen, resulting in the creation of thermal NOx. Higher adiabatic flame temperature of biodiesel leads to the higher nitrogen oxide emission. The maximum increases of NO_x_ concentration for B5, B10 and B20 were 2.5, 5, 7.5 and 10%, respectively compared to diesel fuel at full load. These results were agreed according to Luján et al.^50^ and Xue et al.^51^.

### Unburned hydrocarbons emission (HC)

**Figure 6:**
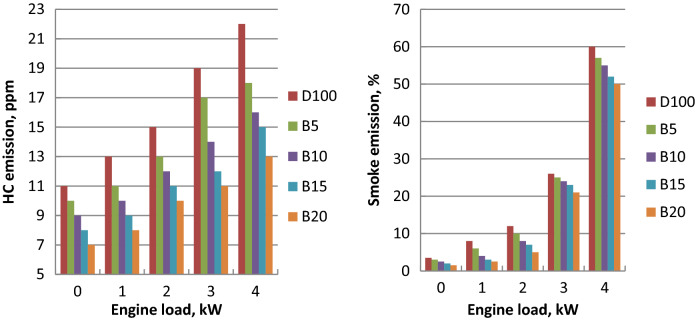
Variations of unburned hydrocarbon and smoke opacity with engine loads.

Figure 6 shows the values of unburned hydrocarbon concentrations of diesel, ethyl esters and their mixtures related to the engine load. HC emissions of ethyl ester blends were less than diesel fuel and it was declined with the ethyl ester ratio increase because of the clean combustion. Biodiesel blends enhanced the oxidation of unburned hydrocarbons due to the oxygen concentration. The cylinder temperature increase of biodiesel was responsible of HC decrease about diesel oil. Higher cylinder temperature of biodiesel helps in preventing condensation of higher hydrocarbon. Palm ethyl ester of higher cetane number emits the less HC emission about diesel oil. The highest decreases of HC emission for B5, B10, B15 and B20 in comparison to diesel oil at all full load were 12, 17, 24 and 35%, respectively. These findings are confirmed with the literature^14,47^.

### Smoke opacity emission

Smoke opacity at different engine brake power for biodiesel blends are described in Fig. 6. For the entire load range, biodiesel blended fuels produced the less smoke than diesel oil. Because of the advanced combustion and higher cetane number, smoke opacity decreased at medium and higher engine loads as the biodiesel percentage increased. The presence of oxygen and reduced rich fuel zone of palm biodiesel lead to the combustion enhancement and smoke emission reduction. The maximum reductions of smoke opacity for B5, B10, B15 and B20 were 5, 8, 13 and 18.5% compared to diesel fuel at 100% of engine load, respectively. These results were agreed with the references^52,53^.

### Cylinder pressure

**Figure 7:**
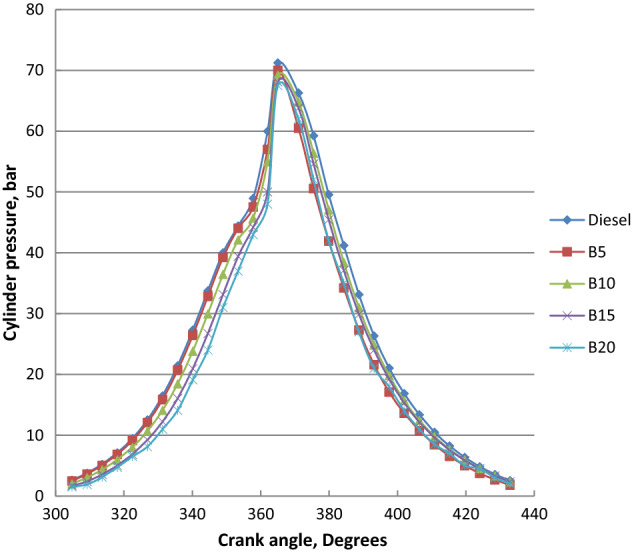
Cylinder pressure at different crank angles and full load for biodiesel blends.

**Figure 8:**
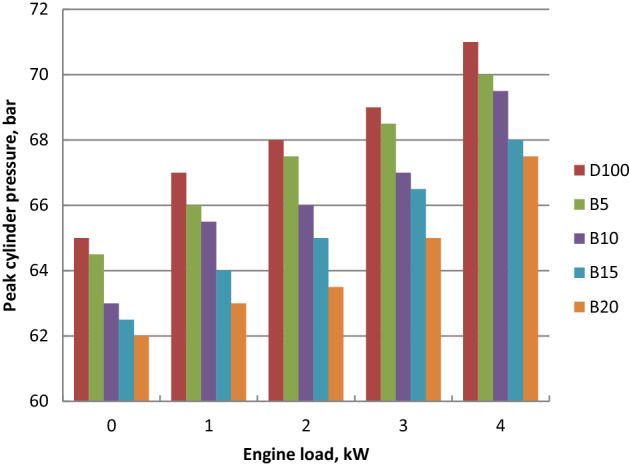
Peak Cylinder pressure at different loads for biodiesel blends.

Cylinder pressures of diesel and ethyl ester blends at different crank angles and full load are summarized in Fig. 7. Peak cylinder gas pressure of biodiesel mixtures were lower in comparison to diesel fuel and it declined with the increase of ethyl ester content in blends. Longer ignition delay of diesel oil led to combustion starts later. It increases as one moves away from TDC during the expansion stroke and is influenced by the consumed fuel during the premixed burning period. Because biodiesel has a shorter ignition delay and higher cetane number burning begins earlier than diesel oil. Premixed combustion heat release and higher pressure rise rate for diesel fuel about biodiesel blends was shown. Biodiesel has a higher bulk modulus, faster sound speed in the fuel injection pipe, and it can be injected earlier. Diesel oil produced 71.22 bar at 90.5° After Top Dead Centre (ATDC). Palm biodiesel blends B5, B10, B15 and B20 showed the peak cylinder pressure of 70, 69.2, 68.1 and 67.5 bar at 89.5, 89.4, 89.3 and 90 ATDC, respectively. Peak cylinder pressure (P_max_) increased with the output power increase because of the more consumed fuel at higher loads as indicated in Fig. 8. Palm ethyl ester blends record lower P_max_. compared to diesel oil. As the content of ethyl ester in the mixtures increased, the peak cylinder pressure decreased. Because of the shorter ignition delay and advanced injection timing, higher density, higher bulk modulus, and higher cetane number, biodiesel has lower peak cylinder pressure. Higher fuel viscosity results in increased fuel penetration, poor atomization, slow air–fuel mixing and decreased cone angle. Biodiesel higher molecular weight and lower laminar burning velocity resulted in insufficient utilization of fuel energy. The decreases in peak cylinder pressure at 4 kW are 1.7, 2.83, 4.38 and 5.22% for B5, B10, B15 and B20, respectively in comparison to diesel fuel. These findings were confirmed with the literature^54,55^.

### Heat release rate (HRR)

**Figure 9:**
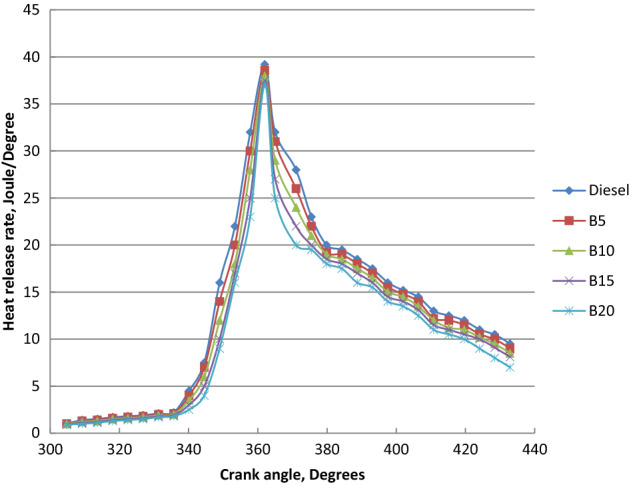
Impact of palm ethyl ester mixtures on HRR at crank angle variation and full load.

Ethyl esters heat release rate for ethyl ester blends with diesel fuel at the engine load variation were shown in Fig. 9. HRR of diesel, palm biodiesel and their blends showed the same trend. Maximum heat release rates for ethyl ester mixtures were declined than diesel fuel. Peak HRR decreased and occurred earlier for ethyl esters blends as the percentage of ethyl ester increased about diesel oil. It is ascribed to the biodiesel higher cetane number and shorter ignition compared to conventional diesel. Earlier combustion initiation of biodiesel reduces the ignition delay about diesel oil. Amount of fuel burned in the premixed combustion phase was lower for biodiesel about diesel oil but more fuel was burned in the diffusion phase. The larger bulk modulus of ethyl ester leads to the higher sound speed in the injection system and the earlier burning of diesel oil. Diesel fuel has greater rate of pressure rise and premixed combustion heat release than biodiesel mixtures. Biodiesel has lower HRR than diesel due to the lower calorific value and shorter ignition delay. The increase in biodiesel concentration resulted in a reduction in heat release during premixed combustion. Increase of fuel accumulation over the longer delay period, producing in the higher heat release rate of diesel oil. Surface tension and inadequate spray atomization of biodiesel reduce the heat release of biodiesel. Due to ethyl ester late burning, ethyl esters blends had greater HRR than diesel fuel in the late phase of combustion. Peak heat release rates of diesel oil, B5, B10, B15 and B20 are 39.2 J/CA, 38.57 J/CA, 38 J/CA and 37.5 J/CA and 37.1 J/CA, respectively at crank angles of 65, 67, 68, 69 and 71 before TDC^14,52–55^.

### Ignition delay period (ID)

**Figure 10:**
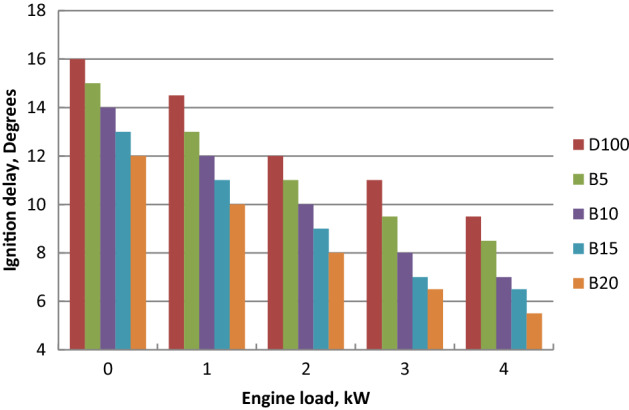
Effect of palm ethyl ester mixtures on ignition delay at engine load variation.

Figure 10 showed the ignition delay profile of all fuels at engine load variation. Higher cetane number of palm ethyl ester blends led to the shorter ignition delay compared to diesel fuel. Ignition delay period decreased for all blends as the output power increased due to the consumed fuel and cylinder temperature increases. Biodiesel blends ignition delays were lower than diesel oil and decreased with biodiesel content increase when compared to diesel oil. Advanced start of injection is because of lower compressibility and high viscosity of biodiesel. ID is affected by cetane number. Shorter ignition delay led to reduction of premixed combustion period. Higher turbulence intensity and cylinder temperature lead to the improvement of fuel–air mixing and vaporization rate. Biodiesel higher viscosity and molecular weight increase the fuel droplet size and vaporization duration. The maximum declines in ignition delay for B5, B10, B15 and B20 at full load (4 kW) were 10, 17, 25 and 30% than diesel fuel, respectively and this confirmed with literature^56^.

### Cylinder temperature

**Figure 11:**
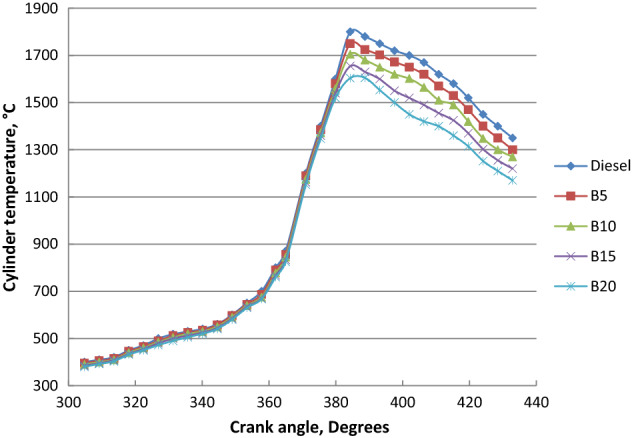
Cylinder temperature at different crank angles and full load for biodiesel blends.

Effects of biodiesel blends on cylinder temperature at different loads were shown in Fig. 11. Cylinder temperature decrease with ethyl ester percentage increase is due to the pressure cylinder decrease and its influence on the premixed combustion stage. Less biodiesel volatility and lower evaporation rate led to the cylinder temperature decrease. The diffusion combustion stage increased as the palm biodiesel percentage was increased. The molecular structure of fatty acid ethyl ester led to the lower final boiling point and faster combustion process. Because of the pressure gradient reduction, the maximum mean cylinder temperature reduced as the biodiesel concentration increased, affecting the premixed combustion stage. Peak cylinder temperatures for diesel fuel and palm biodiesel blends B5, B10, B15 and B20 are 1820, 1783, 1767, 1745 and 1720 K, respectively.

### Combustion duration

**Figure 12:**
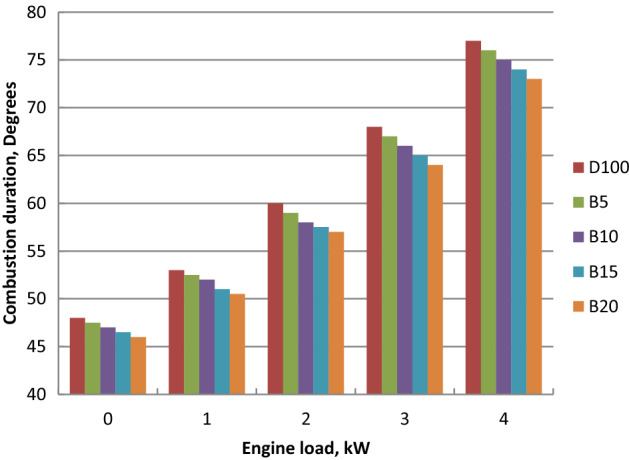
Combustion durations with engine load variation for biodiesel blends.

Influence of ethyl ester mixtures on combustion duration at engine loads variations was shown in Fig. 12. It was increased as the amount of fuel injected increased, resulting in an increase in brake power. Combustion duration is shorter for biodiesel in comparison to diesel oil. As palm biodiesel percentage increased, faster combustion led to the combustion duration reduction. Molecular structure of palm ethyl esters and its lower boiling point led to the faster combustion process^56^. Combustion duration values for diesel oil are 76.41ºCA, while the values are 75.6, 74.24, 73.66 and 72.58ºCA for B5, B10, B15 and B20, respectively at full load.

### Artificial neural network modeling

Artificial neural network (ANN) is used to predict the engine performance, emissions and combustion characteristics. Engine load and the ethyl ester concentration in the blends are two input variables (engine load, biodiesel percentage) and eleven output variables (BSFC, BTE, exhaust gas temperature, air–fuel equivalence ratio, HC, CO, NO_x_, smoke emissions, peak cylinder pressure, combustion duration and ignition delay). The correlation coefficient (R^2^) of 0.99 shows the successful engine performance, emissions and characteristics prediction.

**Figure 13:**
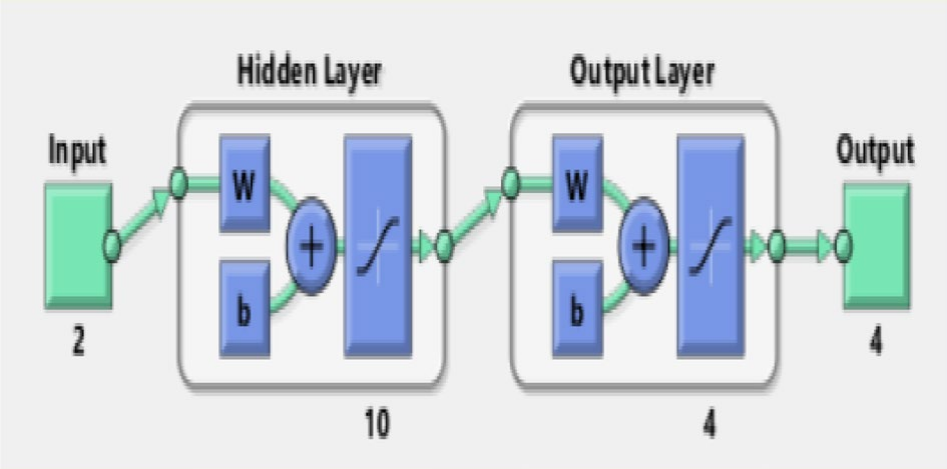
Schematic diagram of ANN model.

In the application of ANN, 70% of the data trials were used for training, 15% for verification, and 15% for testing. ANN was built using the feed-forward back propagation network type, Logsig transfer function, Levenberg–Marquardt (Trainlm) function, and learning function (LEARNGDM). The feed-forward back propagation network was used to describe complex problems in system modeling and identification. Table 3 and Fig. 13 show the neural network construction of the ANN model. There are three stages to ANN prediction. The first stage consists of introducing the input parameters. By analyzing the validation, training, and test data, the ANN was trained with these inputs to produce the best trained ANN. Following that, ANN accuracy control was used^44^. Design of experiments techniques, such as RSM (response surface methodology), Taguchi method, and factorial design, can be used to determine the optimum fuel blend ratios based on engine operating parameters. RSM is used to simulate nonlinear relationships between input factors and outputs or responses. For modeling linear relationships, factorial design is used, and the Taguchi method determines only the best combination of factors for the previously determined factor levels at the start of the investigation^58^.

**Table 3:** Neural network construction.

Data	70% of the data trials were used for training, 15% for verification, and 15% for testing
Network type	Feed-forward back propagation
Training function	Trainlm
Learning function	Learngdm
Transfer function	Logsig
Performance function	Mean Square Error (MSE)

**Figure 14:**
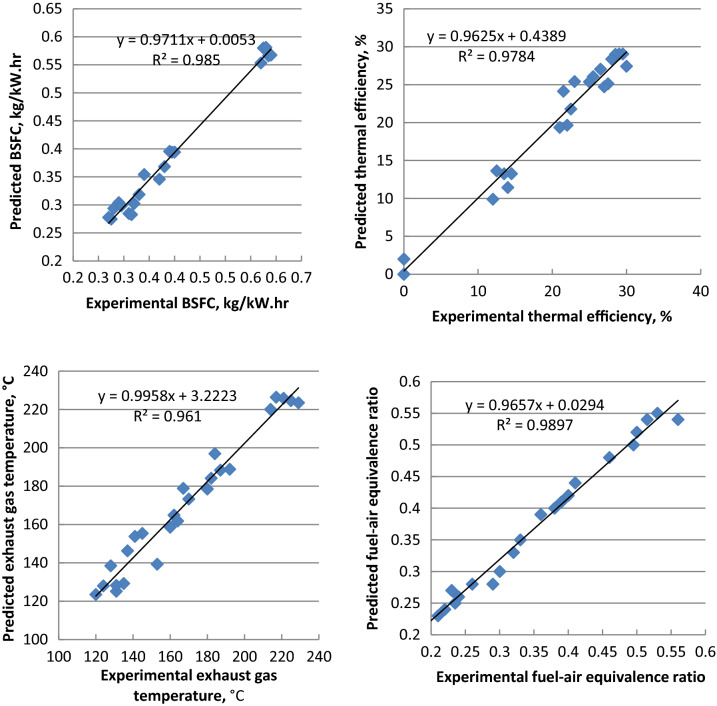
Correlation between ANN model and experimental values of BSFC, BTE, T_exh_ and fuel- air equivalence ratio.

**Figure 15:**
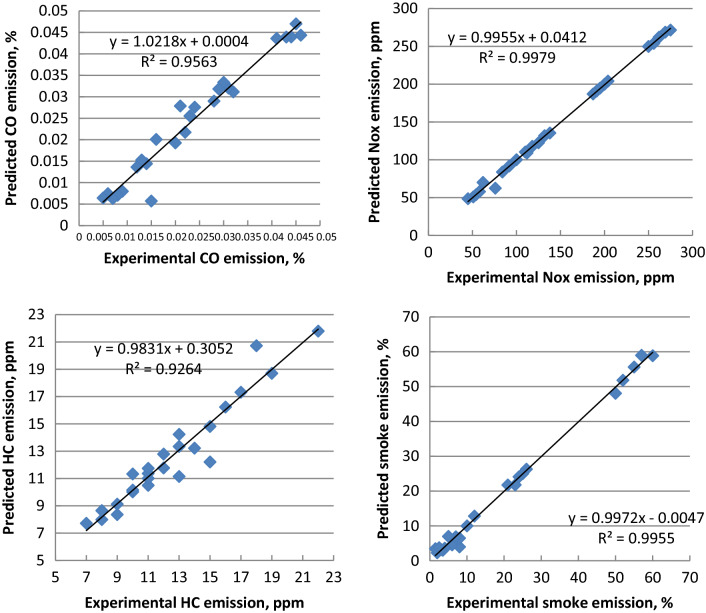
Correlation between ANN model and experimental values of CO, NO_x_, HC and smoke emissions.

**Figure 16:**
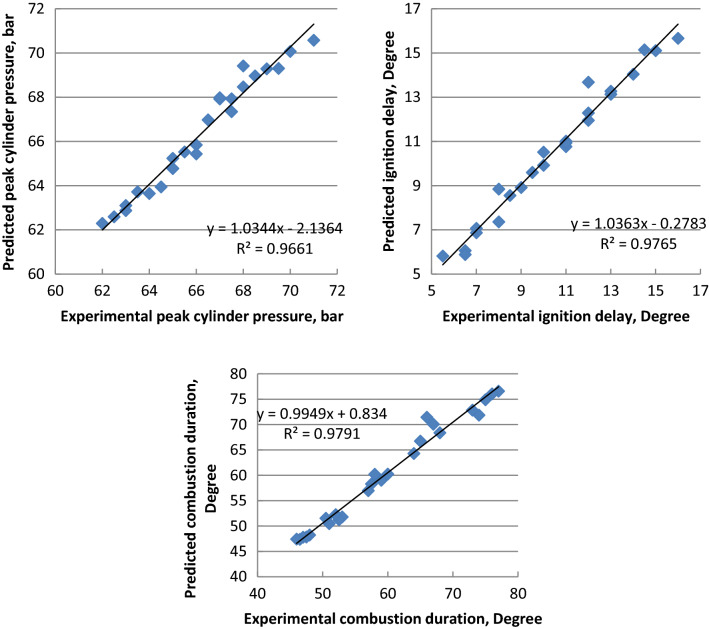
Correlation between ANN model and experimental values of peak cylinder pressure, ignition delay and combustion duration.

ANN is simply interconnections of many neurons which arranged in three layers. First is the input layer data, secondly the hidden layer, and finally the output layer. Figures 14, 15 and 16 indicate that the R^2^ value of BSFC, BTE, exhaust gas temperature, air- fuel equivalence ratio, HC, CO, NO_x_, smoke emissions, peak cylinder pressure, ignition delay and combustion duration values are 0.985, 0.9784, 0.961, 0.9897, 0.9264, 0.9563, 0.9979, 0.9955, 0.9661, 0.9765 and 0.9791 respectively from the model of ANN. If the value of R^2^ is close to 1 and this leads to better results from the model. The best fit is obtained with NO_x_ emission. The best fit is shown by the highest value of R^2^. The prediction model is accurate and reliable with measured data^36–39^. The mathematical model's accuracy was determined by comparing the actual experimental results to the mathematical model output values. When the difference between the output values and the actual experiments was smaller, the accuracy increased^57,58^.

A comparative analysis between numerical and experimental data at full load condition which shows BTE, BSFC, EGT, AFR, CO, NO_X_, HC, Smoke, Peak cylinder Pressure, peak HRR, Ignition delay and combustion duration was shown in Table 4. This table shows the differences between experimental and numerical values to show the error.

**Table 4:** Comparative analysis between numerical and experimental data at engine full load.

Parameter	Experimental					Numerical				
	D100	B5	B10	B15	B20	D100	B5	B10	B15	B20
BSFC (kg/kW.hr)	0.27	0.275	0.28	0.29	0.295	0.277	0.275	0.294	0.304	0.299
BTE (%)	30	29.5	29	28.5	28	27.43	29.04	29.03	28.99	28.37
EGT (°C)	214	217	221	225	229	220.03	226.43	225.83	224.59	226.19
AFR	0.56	0.53	0.515	0.5	0.495	0.192	0.231	0.369	0.478	0.491
CO emission (%)	0.046	0.045	0.044	0.043	0.041	0.0152	0.0293	0.0422	0.0442	0.0414
NO_x_ emission (ppm)	250	256	262	269	275	249	254	262	268	271
HC emission (ppm)	22	18	16	15	13	22.34	21.22	16.72	15.1	14.2
Smoke emission (%)	60	57	55	52	50	58.87	58.97	55.63	51.82	49.32
Peak cylinder pressure (bar)	71	70	69.5	68	67.5	70.57	70.07	69.3	68.47	67.43
Peak HRR (J/Degrees)	39.2	38.57	38	37.5	37.1	38.7	38.1	37.6	37.1	36.7
Ignition delay (Degrees)	9.5	8.5	7	6.5	5.5	9.6	8.6	7.07	5.89	5.92
Combustion duration (Degrees)	77	76	75	74	73	76.58	76.04	74.89	71.84	72.83

## Conclusions

Palm ethyl ester was produced from its oil and its properties were agreed with ASTM standards. A single cylinder C.I.E. was run with diesel and palm biodiesel mixtures (5, 10, 15 and 20%) at different engine loads. Performance, emissions and combustion characteristics were in comparison to diesel oil. The most important points are summarized as follows:

- The maximum decreases in thermal efficiency, fuel–air equivalence ratio for B20 were 1.5, 3.5, 6 and 8% but the maximum increases in BSFC, exhaust gas temperature and NO_x_ emission for B20 at full load about diesel fuel were 9, 8 and 10%, respectively.
- The highest decreases in CO, HC and smoke emissions of B20 about diesel oil at full load were 2, 35 and 18.5% at full load, respectively.
- Maximum decrease in peak cylinder pressure at full load is 5.22% for B20 about diesel oil. Peak heat release rates of diesel oil, B5, B10, B15 and B20 are 39.2 J/CA, 38.57 J/CA, 38 J/CA and 37.5 J/CA and 37.1 J/CA, respectively. The maximum decline in ignition delay for B20 at full load (4 kW) is 30% than diesel fuel.
- Maximum cylinder temperature for diesel fuel and palm ethyl ester B20 is 1720 K. The combustion durations are 76.41, 75.6, 74.24, 73.66 and 72.58 ºCA for diesel oil, B5, B10, B15 and B20, respectively at full load.
- ANN is a valuable technique used to predict the engine performance, emissions and combustion characteristics. The R^2^ value of BSFC, BTE, T_exh_, air- fuel equivalence ratio, HC, CO, NO_x_, smoke emissions, peak cylinder pressure, ignition delay and combustion duration values are 0.985, 0.9784, 0.961, 0.9897, 0.9264, 0.9563, 0.9979, 0.9955, 0.9661, 0.9765 and 0.9791 respectively from the model of ANN. The prediction model is accurate and reliable with measured data. The prediction model is accurate and reliable with measured data.
- Produced palm biodiesel is friendly and showed improved performance, combustion and emissions reductions in comparison to diesel fuel and could be applicable up to 20% in diesel engine.

## Data Availability

All data are available within the published paper.

## Abbreviations

ANN: Artificial neural network
BSFC: Brake specific fuel consumption, kg/kW.hr
BTE: Brake thermal efficiency, %
B5: Biodiesel 5% + Diesel 95% by volume
B10: Biodiesel 10% + Diesel 90% by volume
B15: Biodiesel 15% + Diesel 85% by volume
B20: Biodiesel 20% + Diesel 80% by volume
CI: Compression ignition
CO: Carbon monoxide emission, %
D100: Pure diesel fuel
HC: Hydrocarbon emission, ppm
HRR: Heat release rate, Joule/Degree
ICE: Internal combustion engines
ID: Ignition delay
NOx: Nitrogen oxides emission, ppm
T_exh__._: Exhaust gas temperature, °C
